# Using Stable Isotope Compositions of Animal Tissues to Infer Trophic Interactions in Gulf of Mexico Lower Slope Seep Communities

**DOI:** 10.1371/journal.pone.0074459

**Published:** 2013-12-06

**Authors:** Erin L. Becker, Erik E. Cordes, Stephen A. Macko, Raymond W. Lee, Charles R. Fisher

**Affiliations:** 1 Department of Biology, Pennsylvania State University, University Park, Pennsylvania, United States of America; 2 Biology Department, Temple University, Philadelphia, Pennsylvania, United States of America; 3 Department of Environmental Sciences, University of Virginia, Charlottesville, Virginia, United States of America; 4 School of Biological Sciences, Washington State University, Pullman, Washington, United States of America; University of Pennsylvania, United States of America

## Abstract

We analyzed the tissue carbon, nitrogen, and sulfur stable isotope contents of macrofaunal communities associated with vestimentiferan tubeworms and bathymodiolin mussels from the Gulf of Mexico lower continental slope (970-2800 m). Shrimp in the genus *Alvinocaris* associated with vestimentiferans from shallow (530 m) and deep (1400-2800 m) sites were used to test the hypothesis that seep animals derive a greater proportion of their nutrition from seeps (i.e. a lower proportion from the surface) at greater depths. To account for spatial variability in the inorganic source pool, we used the differences between the mean tissue δ^13^C and δ^15^N of the shrimp in each collection and the mean δ ^13^C and δ^15^N values of the vestimentiferans from the same collection, since vestimentiferans are functionally autotrophic and serve as a baseline for environmental isotopic variation. There was a significant negative relationship between this difference and depth for both δ^13^C and δ^15^N (p=0.02 and 0.007, respectively), which supports the hypothesis of higher dependence on seep nutrition with depth. The small polychaete worm *Protomystides* sp. was hypothesized to be a blood parasite of the vestimentiferan *Escarpialaminata* . There was a highly significant linear relationship between the δ^13^C values of *Protomystides* sp. and the *E* *. laminata* individuals to which they were attached across all collections (p < 0.001) and within a single collection (p = 0.01), although this relationship was not significant for δ^15^N and δ^34^S. We made several other qualitative inferences with respect to the feeding biology of the taxa occurring in these lower slope seeps, some of which have not been described prior to this study.

## Introduction

The expansion of oil and natural gas exploration and extraction into ever-deeper waters of the Gulf of Mexico increases the potential threat to the unique animal communities that thrive in natural oil and gas seep environments on the deep continental slope. We still lack some of the most basic knowledge about deep-sea animals, their life histories and interspecific interactions, their role in the wider Gulf of Mexico food web, and how anthropogenic disturbance will affect their long-term survival.

**Figure 1:**
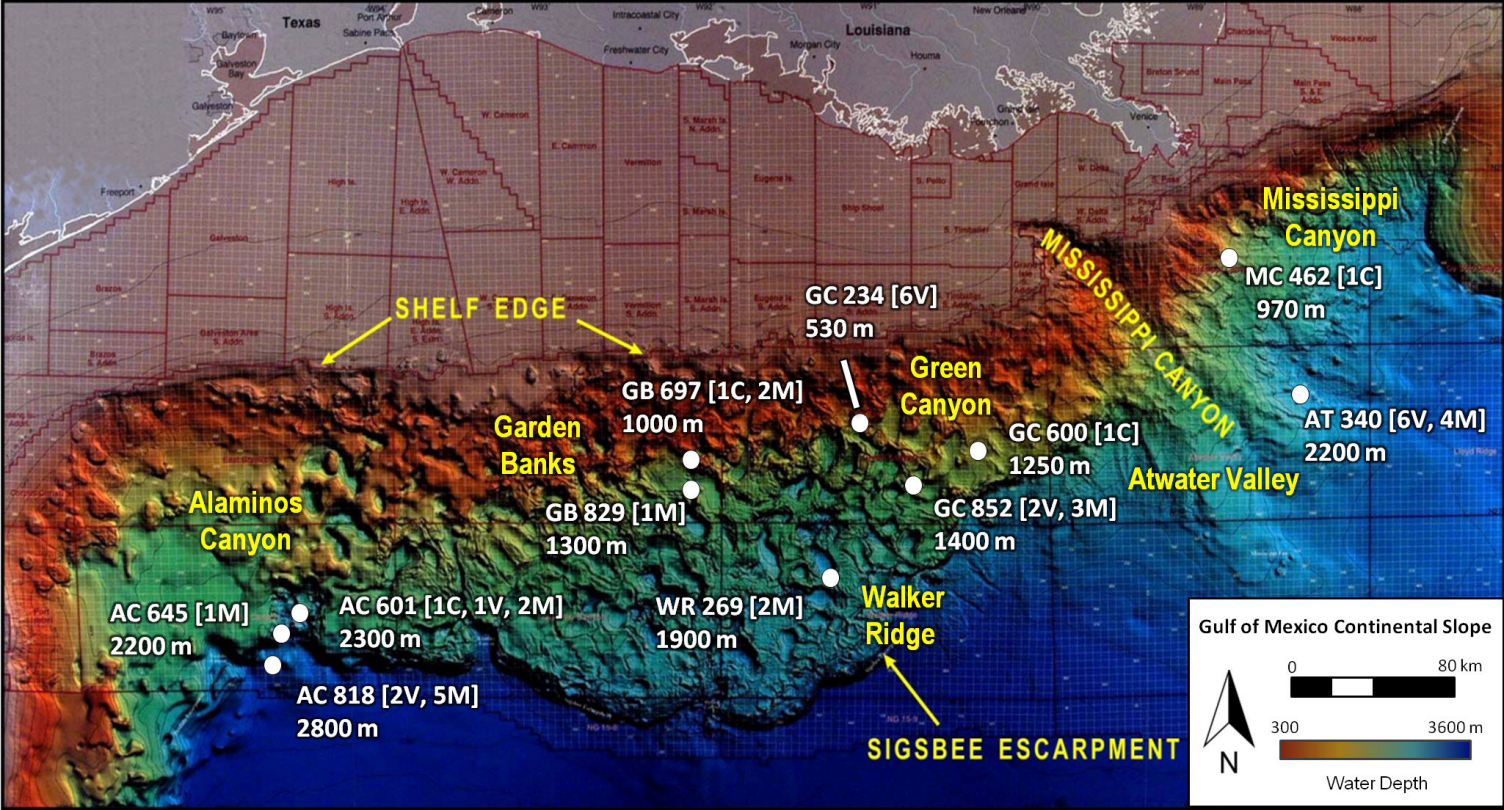
Map of collection sites on the Gulf of Mexico lower continental slope. The site names are based on Bureau of Ocean Energy Management (BOEM) lease block designations and consist of a two-letter abbreviation, which stands for the region (AC=Alaminos Canyon, for example), followed by a three-digit number. The yellow text gives the name for the region, while the white points and text signify the specific study sites. The notations inside the brackets indicate how many of each community type was collected at a site: C=vesicomyid clams ( *Calyptogena* *ponderosa* and an undescribed vesicomyid), M=mussels ( *Bathymodiolus* spp.), and V=vestimenitiferans ( *Escarpialaminata* and *Lamellibrachia* spp.). The bathymetric depth is given in meters below the site name.

Trophic interactions are one of the most basic components of an animal’s or community’s ecology, but owing to the inherent difficulties in studying deep-sea ecosystems, chemosynthesis–based seep food webs are not very well-constrained. This is especially true for seeps occurring in deeper water, which are historically less well-studied than shallower seeps. During two expeditions in 2006 and 2007, a multidisciplinary team of researchers worked to discover, explore, and sample seep communities on the deep slope, covering a large geographic and bathymetric range (700 km east to west and from 970 to 2800 m depth) (Figure **1**). This effort has greatly increased our understanding of the geochemistry, community composition, species distributions, nutritional sources, geology, and population genetics of deep seep communities [1].

In this study, we used stable isotopes as a tool to elucidate the trophic structure of animal communities at these deep seep sites. The community composition of seeps in the deeper Gulf of Mexico differs significantly from shallower communities at high taxonomic levels [2], and there is little published work about the feeding biology of most of the taxa occurring in deeper water. Stable isotope analysis is a valuable tool in the deep sea where direct observation and gut content analysis are difficult (many animals are very small and some thoroughly grind their food) and has led to some of the most significant discoveries about the nutrition of cold seep and related hydrothermal vent animals [3,4,5,6].

We made quantitative collections of the communities associated with two dominant seep taxa: vestimentiferan tubeworms and bathymodiolin mussels. These taxa dominate biomass at seeps, contain symbiotic bacteria that provide their nutrition, and provide habitat for a community of smaller animals. Although there is some overlap in the species associated with vestimentiferans and mussels, there are significant differences in species composition of the associated communities and, due to differences in their symbionts and life histories, they inhabit very different geochemical habitats [2]. Mussels have methanotrophic symbionts in their gills and therefore live in active seep sites where methane is present in the bottom water above the sediment surface, while vestimentiferans have chemoautotrophic sulfide-oxidizing symbionts and can live in less active locations because of their ability to mine sulfide from the sediment through their “roots” [7]. In total, we made 11 vestimentiferan and 20 mussel community collections from 970 to 2800 m depth (see also 8). Additionally, data were included from vestimentiferan communities collected at 530 m depth for comparison with deeper sites [9].

The extensive sampling allowed us to discern qualitative patterns and make inferences about the feeding biology of seep animals. We also tested the long-standing hypothesis that generalist seep animals (those that obtain nutrition from both surface and seep primary production) derive a greater proportion of their nutrition from seeps as depth increases. This hypothesis is based on the fact that organic material produced by photosynthesis at the surface is consumed and degraded as it sinks toward the bottom, and thus, less of it is available for consumption at greater depths. This is supported by a general trend of decreasing benthic biomass with increasing depth in the “normal” deep sea [10], but no data exist that support this trend for vents and seeps, where primary production occurs locally via chemosynthesis.

We tested this hypothesis by comparing the stable isotope compositions of alvinocarid shrimp from shallow and deep vestimentiferan communities to determine whether there is isotopic evidence of greater usage of seep-derived nutrition at greater depths. Organic matter from the photic zone has δ^13^C values between -23 and -19‰ [11], and carbon fixed by chemoautotrophy ranges from -75 to -28‰ [12,13], with the more negative values in this range indicating carbon derived from methane, which itself can vary depending on whether it is of biogenic (δ^13^C ^≈^ -80 to -60‰) or thermogenic (δ^13^C ^≈^ -55 to -30‰) origin [14]. δ^15^N values in surface-derived organic material are generally greater than 6‰ [15], while seep animals can have δ^15^N that are much lower and even negative [13]. Thus, a trend of decreasing δ^13^C and δ^15^N values with depth could indicate increased usage of seep-derived nutrition.

**Figure 2:**
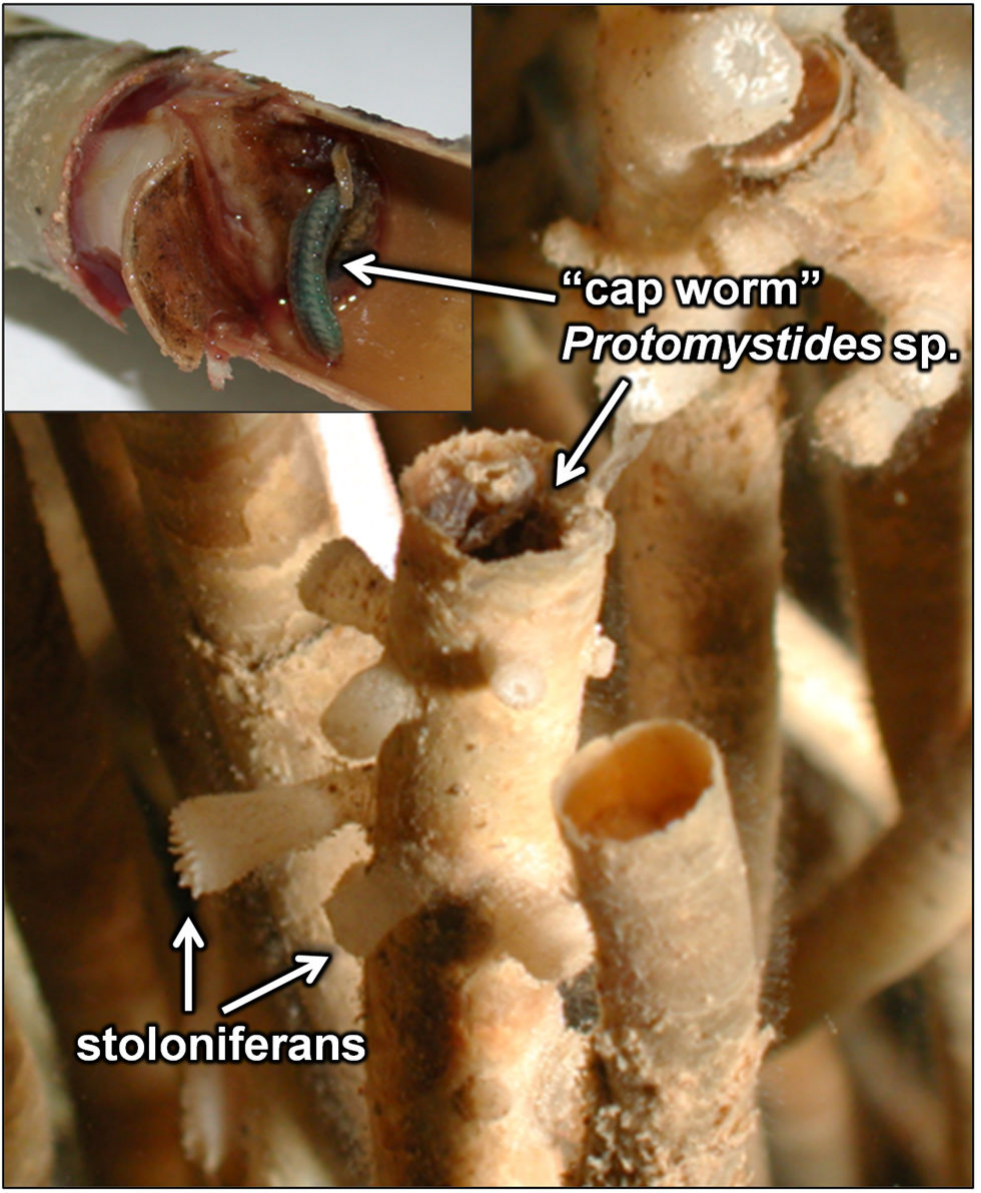
A close-up photo of *Protomystides* sp. inhabiting its casing atop the obturaculum of *Escarpialaminata* . Stoloniferans are also shown attached to the anterior portions of vestimentiferan tubes.

Two hypotheses of specific trophic relationships were also tested. While collecting vestimentiferan communities, clusters of the polychaete “cap worm” *Protomystides* sp. were observed living within a matrix of tubes affixed to the tops of the vestimentiferan *Escarpialaminata* (Figure **2**). The guts of these worms were filled with a red substance resembling tubeworm blood. It was therefore hypothesized that *Protomystides* sp. is a blood parasite of *E* *. laminata* , and we tested for isotopic evidence of this trophic relationship. A polynoid *Branchipolynoeseepensis* lives in the gills of the seep mussel *Bathymodiolusheckerae* , and a nautilliniellid polychaete also lives in the gills of *B* *. heckerae* and vesicomyid clams ( *Calyptogena* *ponderosa* and an undescribed vesicomyid). We looked for stable isotope evidence of a trophic relationship between the commensals and their bivalve hosts, as was previously found for *Branchipolynoesymmytilida* and the hydrothermal vent mussel *Bathymodiolus* *thermophilus* [16].

## Methods

### Study sites

The study sites are named according to the Bureau of Ocean Energy Management lease block designations. Each name includes a two-letter abbreviation for the region (e.g. GC for Green Canyon) followed by a three-digit number. The 13 sites in this study are located along the lower continental slope of the Gulf of Mexico from 225 km south of Texas near the Texas-Louisiana border to the south of Alabama (Table **1**, Figure **1**). Sites ranged in depth from 970 m to 2800 m. Descriptions of the study sites are given in [17] and [18]. Data was also included from a site at 530 m for comparison between shallow and deep sites (Figure **1**).

**Table 1:** Information for each community collection whose stable isotope data are displayed in Figures 5, 6 and 7.

**Figure**	Collection ID*	**Sample**	**Dive**	**Latitude**	**Longitude**	**Depth**
**5A**	GB829m1	mpa	J2-279	27:11.1	-92:07.5	1258
**5B**	GC852m1	mp	4186	27:06.3	-91:09.9	1410
**5C**	GC852m5	mpa	J2-278	27:06.6	-91:09.9	1407
**5D**	GC852m6	mpf	J2-278	27:06.3	-91:09.9	1408
**5E**	GB697m1	mp	J2-274	27:18.7	-92:06.3	1005
**5F**	GB697m2	ms	J2-274	27:19.2	-92:06.6	1015
**5G**	WR269m3	mpf	J2-275	26:41.1	-91:39.7	1910
**5H**	WR269m4	ms	J2-275	26:41.1	-91:39.7	1910
**6A**	AC601m1	mpb	J2-283	26:22.1	-94:31.1	2284
**6B**	AC601m2	mpd	J2-283	26:22.1	-94:31.1	2284
**6C**	AC645m4	mpb	J2-281	26:21.2	-94:29.8	2197
**6D**	AC818m1	mp	4192	26:10.8	-94:37.3	2744
**6E**	AC818m2	ms	4195	26:10.8	-94:37.3	2745
**6F**	AC818m3	mpb	J2-282	26:10.8	-94:37.4	2745
**6G**	AC818m4	mpd	J2-282	26:10.8	-94:37.4	2744
**6H**	AC818m5	mpd	J2-284	26:10.8	-94:37.3	2745
**6I**	AT340m1	ms	4180	27:38.6	-88:21.8	2183
**6J**	AT340m2	mp	4181	27:38.8	-88:22.2	2199
**6K**	AT340m-	ms1,ms2	J2-270	27:38.7	-88:21.9	2190
**6L**	AT340m-	mpa,mpf	J2-277	27:38.8	-88:21.8	2190
**7A**	AC601t1	bm	4196	26:23.4	-94:30.8	2323
**7B**	AC818t1	bm	4195	26:10.8	-94:37.3	2745
**7C**	AC818t2	bm	J2-282	26:10.7	-94:37.2	2746
**7D**	AT340t1	bm	4179	27:38.6	-88:21.8	2185
**7E**	AT340t2	bm	4180	27:38.6	-88:21.8	2184
**7F**	AT340t3	bm	4183	27:38.8	-88:22.4	2179
**7G**	AT340t4	bm	J2-270	27:38.6	-88:21.8	2192
**7H**	AT340t5	bm	J2-276	27:25.1	-88:21.8	2188
**7I**	AT340t6	bm	J2-277	27:38.8	-88:22.4	2175
**7J**	GC852t1	bm	4186	27:06.3	-91:09.9	1409
**7K**	GC852t-	bm	J2-273	27:06.4	-91:09.9	1410

Sample types are Bushmaster (bm), mussel pot (mp), or mussel scoop (ms). Dive numbers are from the *DSV Alvin* (41##) or *ROV Jason II* (J2-###).

* The majority of these samples were analyzed for community composition in [2] and were already given ID numbers consisting of the site name followed by “t” or “m” for a vestimentiferan (tubeworm) or mussel collection, respectively, and a sequential number. Collections with “t- ” or “m- ” after the site name were not included in [2], because they were not quantitative samples.

### Habitat-forming fauna

Vestimentiferans and bathymodiolin mussels are the most common and abundant symbiotic taxa in cold seeps on the Northern Gulf of Mexico lower slope. Here, there are three vestimentiferan species: *Escarpialaminata* and *Lamellibrachia* sp. 1 are common and frequently co-occur in the same aggregations, and *Lamellibrachia* sp. 2 is rare and occurs with the other two [19]. The δ^13^C and δ^34^S values of co-occurring species were not found to be significantly different, but δ^15^N values in *E* *. laminata* were consistently more enriched than *Lamellibrachia* sp. 1 by 2.6 ± 0.7‰ [20]. All known vestimentiferans contain sulfide-oxidizing chemoautotrophic endosymbionts. The associated community inhabits the chitinous tubes and interstices of vestimentiferan aggregations, and older worms can extend up to a meter above the sediment surface, providing habitat with very little exposure to seeping fluids [7,21]. In very old vestimentiferan aggregations on the upper slope, seepage may be undetectable even at the sediment surface [21], and the vestimentiferans survive by mining sulfide from deep in the sediment with their buried “roots” [7].

The three bathymodiolin mussel species that occur on the lower slope are *Bathymodioluschildressi* , which is also common in shallower seeps (overall depth range 525 to 2284 m and collected between 1005 and 2284 m in this study), *B* *. brooksi* (collected between 1080 and 2745 m), and *B* *. heckerae* (collected between 2180 and 2745 m) [2]. Where the depth ranges of these species overlap, *B* *. brooksi* often co-occurs in the same aggregations with either *B* *. childressi* or *B* *. heckerae* , but the latter two species have never been found to co-occur. *B* *. childressi* has only methanotrophic symbionts [22], *B* *. brooksi* forms a dual symbiosis with both chemoautotrophic and methanotrophic bacteria [23], and *B* *. heckerae* contains four different symbionts: a methanotroph, two chemoautotrophs, and one methylotroph-related phylotype [24]. The associated community inhabits the shells of mussels and interstices between them. Mussel beds can be many layers thick, and because mussels lack binding proteins to transport molecules to their symbionts, they require sufficient concentrations of seep fluid and oxygen in the epibenthic water to support autotrophy.

A third, less commonly observed symbiotic taxon at Gulf of Mexico seeps is the vesicomyid clams, which contain sulfide-oxidizing chemoautotrophic endosymbionts [25]. The taxa we collected on the lower slope were *Calyptogena* *ponderosa* and an undescribed vesicomyid. In the Gulf of Mexico, vesicomyids are typically found burrowing through the sediment, leaving distinctive trails. It has been hypothesized that since they acquire sulfide through their foot, the clams must move around when there is not sufficient flux to replenish the sulfide in one location [25]. In this study, we analyzed the stable isotope contents of the clams and their commensal nautilliniellid polychaetes.

### Community collections

Collections were made in 2006 using the deep submergence vehicle *Alvin* and 2007 using the remotely operated vehicle *Jason II*. Quantitative collections of vestimentiferan and mussel communities were obtained using the Bushmaster Jr. and mussel pot or mussel scoop collection devices, respectively [2]. The Bushmaster Jr. is a hydraulically actuated collection device with an open diameter of 0.7 m and lined with a 63-µm mesh [26,27]. The device is placed over an aggregation of vestimentiferans and then closed to capture the vestimentiferans and all animals associated with the vestimentiferan tubes and interstices. This same collection technique was used to collect the vestimentiferan communities from shallower seeps at the GC234 site. Some of the data from these collections that we included in the analysis of alvinocarid shrimp stable isotope contents vs. depth have been published previously [9], and some are from unpublished data.

The mussel pot device as modified from the design of Van Dover (2002) was used to collect mussels [2,28]. This cylindrical aluminum pot is 25 cm in diameter, 30.5 cm in height, and has an internal volume of 0.015 m^3^. The submersible or ROV’s manipulator places the device over a clump of mussels and turns a handle one full rotation to close a Vectran^TM^ skirt. Because some of the mussel communities contained very large mussels that were not effectively captured with the mussel pot, a “mussel scoop” was used to collect some of the community samples [2]. The mussel scoop is a coarse mesh net with an opening of approximately 700 cm^2^ lined with a pillowcase. The mussel communities collected with the two different sampling devices were not significantly different in composition [2]. The manipulator of the submersible dragged the scoop through the mussel bed then placed the entire scoop into an insulated biobox and closed the lid. Vesicomyid clams were sampled using the mussel scoop (AC601 collection) or the submersible’s manipulator (GC600 collection).

Once onboard the ship, Bushmaster Jr. collections were processed for community composition as in [2]. Associated fauna were rinsed from tubeworm tubes, passed through a 1-mm sieve, sorted, and identified to the lowest possible taxonomic level. Up to three individuals of each taxon from each collection were sampled for stable isotope analysis. Tissue samples were obtained from associated fauna by dissecting a piece of muscle tissue from large animals or using whole individuals for smaller animals. The samples were rinsed with deionized water to remove any residual seawater and frozen at -70°C. For the vestimentiferans, vestimentum (muscle) tissue was sampled from up to six individuals of each species from each collection.

Mussel pot and mussel and clam scoop collections were handled similarly. All mussels and clams in these collections were opened to check for commensal polynoids ( *Branchipolynoeseepensis* ) and nautilliniellid polychaetes, and mantle tissue was sampled from up to six individuals of each species from each collection.

We were notified by NOAA through a Letter of Acknowledgement (LOA) that no permissions were needed for our limited collections of invertebrates from the deep Gulf of Mexico for research purposes. This research did not involve any endangered species.

### Stable isotope analysis

All samples were dried at 60°C, homogenized, and acidified to remove inorganic carbonate. Samples were redried and subsamples were analyzed for stable carbon and nitrogen isotopes at the Stable Isotope Facility at the University of California, Davis, using an Integra elemental analyzer coupled with a PDZ Europa 20-20 isotope ratio mass spectrometer (Sercon Ltd., Cheshire, United Kingdom) or by RWL (School of Biological Sciences, Washington State University) using continuous-flow isotope ratio mass spectrometry with a Costech elemental analyzer coupled to a Micromass Isoprime isotope ratio mass spectrometer (EA/IRMS). Data from each of the laboratories are calibrated to NIST (National Institute of Standards and Technology) reference materials. All stable sulfur isotope analysis was performed by SAM at the University of Virginia Stable Isotope Laboratory using continuous-flow isotope ratio mass spectrometry with a Carlo Erba elemental analyzer coupled to a Micromass Optima isotope ratio mass spectrometer (EA/IRMS).

Values are expressed using δ (delta) notation and reported in units of permil (‰), where

$$\begin{array}{c} \delta \text{X = [(Rsample/Rstandard) }-\text{ 1] }\times {\text{ 10}}^{\text{3}}\text{,}\\ {\text{X = }}^{\text{13}}{\text{C, }}^{\text{15}}{\text{N, or }}^{\text{34}}{\text{S and R = }}^{\text{13}}{\text{C/}}^{\text{12}}{\text{C, }}^{\text{15}}{\text{N/}}^{\text{14}}{\text{N, or }}^{\text{34}}{\text{S/}}^{\text{32}}\text{S}\text{.} \end{array}$$

Values are reported relative to PDB (Pee Dee Belemnite) for carbon, air N_2_ for nitrogen, and CDT (Canyon Diablo Triolite) for sulfur.

### Statistical Analysis

To test whether alvinocarid shrimp collected with vestimentiferans show isotopic evidence for incorporating more seep-derived nutrition at greater depths, we conducted a regression analysis in which each data point represents a single collection and the y-component is the difference δ^13^C_shrimp_ -δ^13^C_vest_ or δ^15^N_shrimp_ -δ^15^N_vest_, where δ^13^C_shrimp_ and δ^15^N_shrimp_ are the mean δ^13^C and δ^15^N values of all shrimp in a single collection, and δ^13^C_vest_ and δ^15^N_vest_ are the mean δ^13^C and δ^15^N values of the vestimentiferans from the same collection, and the x-component is the depth at which the vestimentiferan community was collected. Alvinocarid shrimp were used for this analysis, because they are only known from seep and vent sites, are the most common and numerically abundant taxon in both shallow and deep sites (the shallow species is *Alvinocarisstactophila* and the deep species is *A* *. muricola* ) and show isotopic evidence of a generalist feeding strategy that incorporates seep and surface-derived nutrition.

Regression analysis was used to test for a linear relationship between the tissue isotope values of the “cap worm” *Protomystides* sp. and the vestimentiferan *Escarpialaminata* . If *Protomystides* sp. is a parasite of *E* *. laminata* , it was expected that the tissue δ^13^C values of the cap worms would be ~1‰ higher than the tissue δ^13^C value of the individual *E* *. laminata* they were attached to and the tissue δ^15^N would be enriched by approximately 3.4‰, following the average enrichment per trophic level [29,30]. A similar analysis was conducted for commensal polychaetes and their chemosymbiotic bivalve hosts, although this was more qualitative due to small sample sizes.

## Results and Discussion

### Evidence for increased reliance on seep primary production with depth

**Figure 3:**
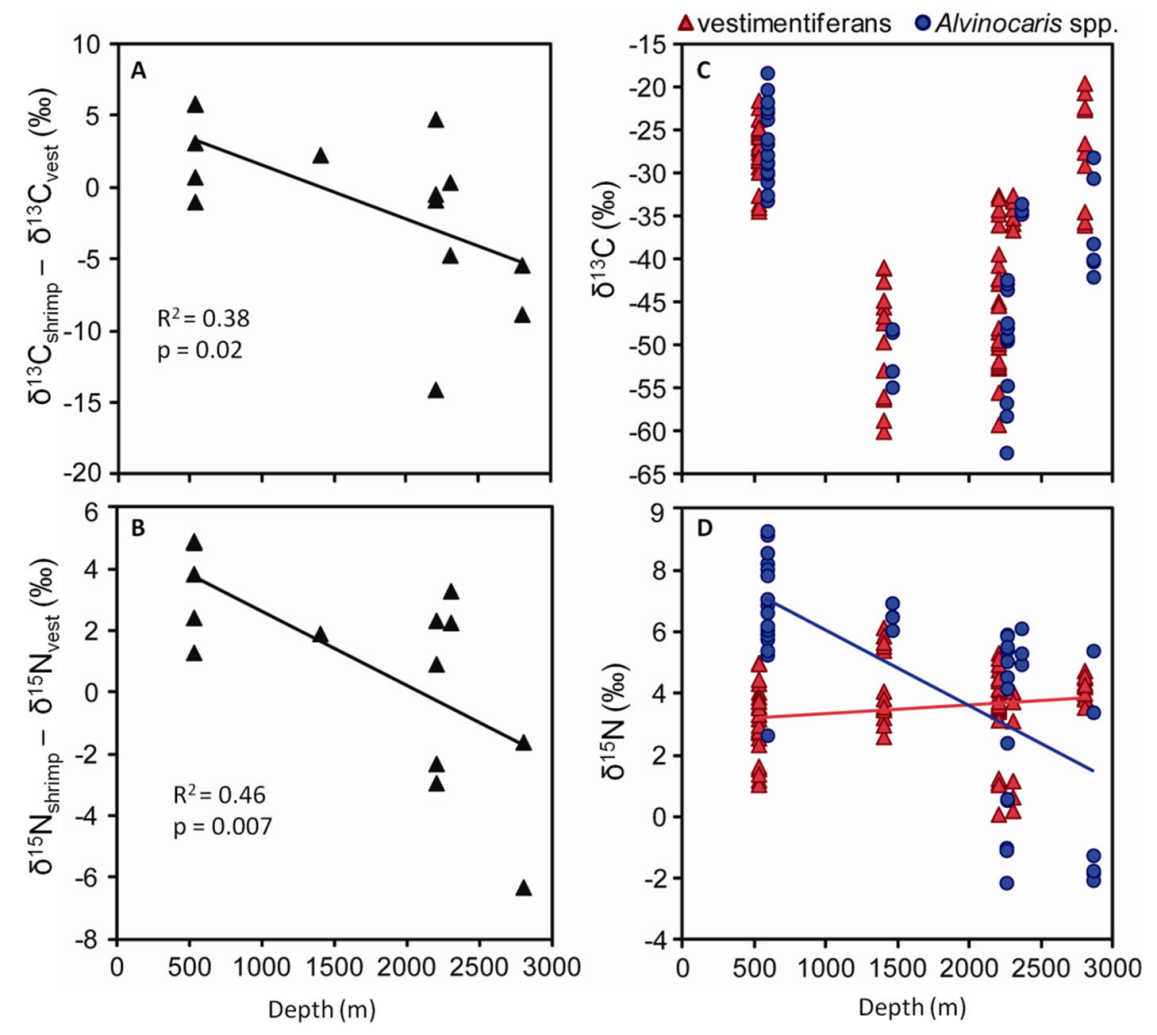
Patterns in tissue δ^13^C and δ^15^N values with depth in *Alvinocaris* shrimp and vestimentiferans. (**A**) The difference between the mean δ^13^C of *Alvinocaris* shrimp (δ^13^C_shrimp_) in each collection and the mean δ^13^C of the vestimentiferan tubeworms in the same collections (δ^13^C_vest_) vs. depth. (**B**) The difference between the mean δ^15^N of *Alvinocaris* shrimp (δ^15^N_shrimp_) in each collection and the mean δ^15^N of the vestimentiferan tubeworms in the same collections (δ^15^N_vest_). (**C**) δ^13^C and (**D**) δ^15^N values for each individual *Alvinocaris* shrimp and vestimentiferan tubeworm sampled vs. depth.

There was a significant negative relationship between δ^13^C_shrimp_ -δ^13^C_vest_ and depth (p = 0.02; Figure **3A**) as well as between δ^15^N_shrimp_ -δ^15^N_vest_ and depth (p = 0.007; Figure **3B**). By examining the patterns of tissue stable isotope values of *Alvinocaris* spp. and vestimentiferans separately, one can see that δ^13^C for both shrimp and vestimentiferans show a U-shaped pattern with depth, though the shrimp generally had higher δ^13^C values than vestimentiferans at shallower depths (some of which were well into the range of photosynthetic production; δ^13^C = -22 to -19‰) and lower δ^13^C values than the vestimentiferans at greater depths, even though the overall δ^13^C values increased from the 2200-2300-m sites to the 2800-m one (Figure **3C**). The δ^15^N values of vestimentiferans remained relatively constant with depth, while the shrimp, albeit variable, showed a trend of decreasing δ^15^N values with depth (Figure **3D**).

**Figure 4:**
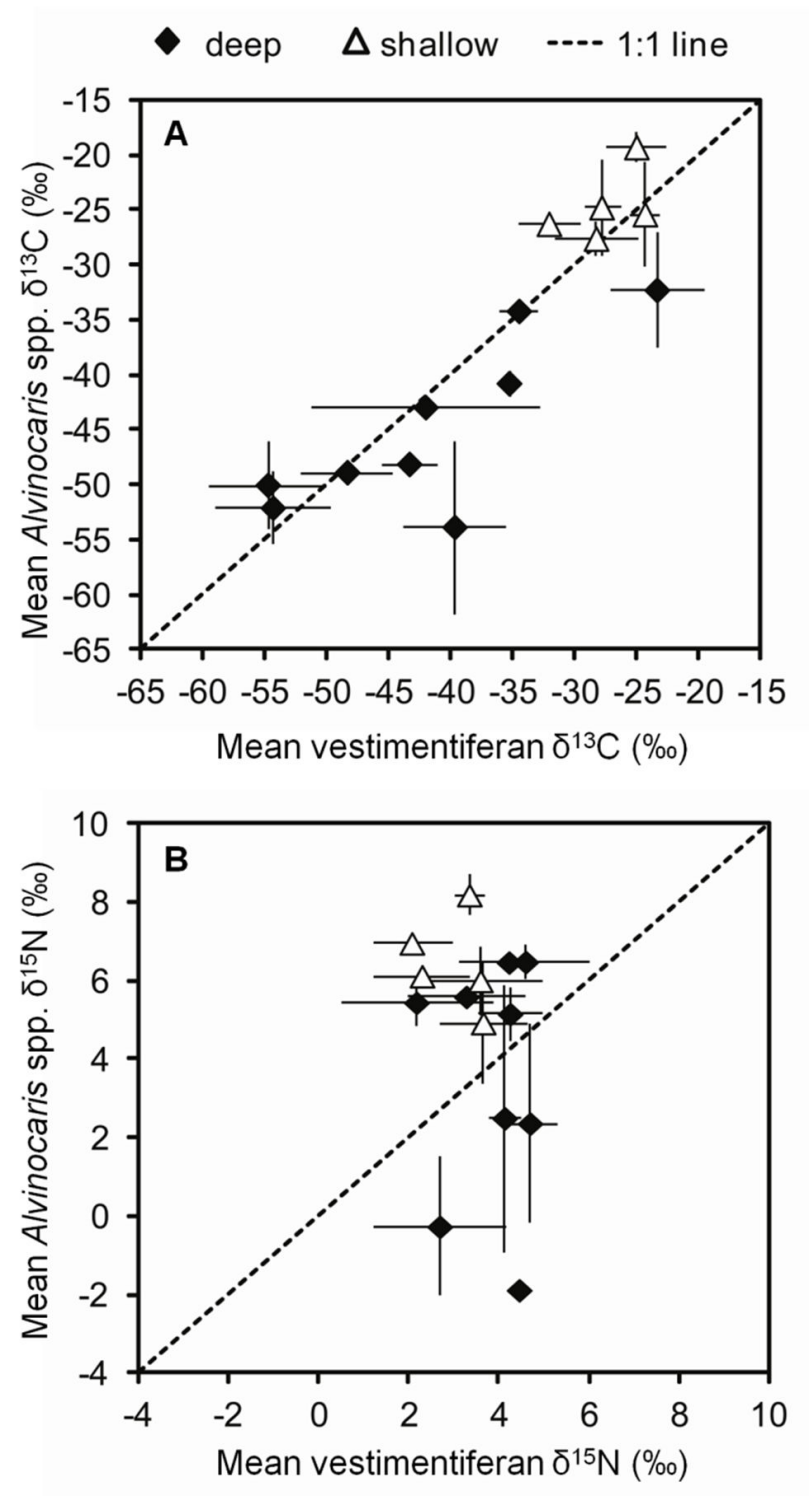
Mean tissue stable isotope contents of *Alvinocaris* spp. vs. vestimentiferans by collection. Mean and standard deviation of (**A**) δ^13^C or (**B**) δ^15^N contents of the shrimp *Alvinocaris* spp. in each collection vs. the mean and standard deviation of the tissue δ^13^C or δ^15^N contents of the vestimentiferan tubeworms in the same collection. “Deep” collections were collected between 1400 and 2800 m depth and contained only *A* *. muricola* , while the “shallow” collections are all from the GC234 study site at 530 m depth and contained only *A* *. stactophila* . The dotted line represents equal values on the x- and y-axes.

The difference between the shrimp tissue stable isotope values and the values of vestimentiferans from the same local environment was calculated, because there is substantial spatial variation in the δ^13^C values of vestimentiferan tubeworms, which can be considered primary producers in this system since they derive the bulk of their nutrition from autotrophic symbionts [20]. This variation is reflected in the associated fauna in general [8] and the alvinocarid shrimps in particular (Figure **4A**), which suggests that there are marked differences in the stable isotope composition of the local dissolved inorganic carbon (DIC) pool. On the other hand, δ^15^N values vary between collections, but there does not seem to be a relationship between the δ^15^N values of vestimentiferans and alvinocarid shrimp, suggesting that their respective variabilities are driven by different processes (Figure **4B**).

These data provide evidence that the shrimp, and potentially other generalist animals, derive more of their nutrition from seeps at greater depths owing to the increasing scarcity of photosynthetically produced material. It is, however, important to acknowledge that there may be other biological or geochemical processes that could lead to this pattern. The shrimp species present at 530 m depth was *Alvinocarisstactophila* , while *A* *. muricola* was the species sampled from the deeper sites between 1400 and 2800 m, so it is possible that the observed trend is due to differences in feeding habits between species. Indeed, if the *A* *. stactophila* data from the shallowest site are removed, the δ^13^C_shrimp_ -δ^13^C_vest_ and δ^15^N_shrimp_ -δ^15^N_vest_ vs. depth relationship becomes statistically non-significant. Also, since sampling was not conducted at different depths within regions (e.g. all 2300 to 2800-m sites are in the AC region, 2200-m in the AT region, and 1000 to 1400-m in the GB and GC regions), we cannot rule out region-specific or depth-related changes in geochemistry as potential causes for the observed pattern. Also, the nitrogen source used by seep vestimentiferans (e.g. ammonia or nitrate) is not known (could be different from that of the shrimp) nor is the δ^15^N contents of the inorganic nitrogen sources at seeps.

Nonetheless, the data presented here show a compelling trend that warrants future investigation, though we remain cautious in interpreting the cause of the observed pattern. In future work, it would be interesting to test this hypothesis using mussel communities from the shallow and deep seeps, since *Alvinocaris* are also abundant in these habitats and, unlike vestimentiferan communities, the tissue δ^15^N contents of mussels and associated communities are apparently affected by the same inorganic nitrogen pool [8], and to sample from sites in the depth range between 530 and 1000 m, since these were not captured in our study. Additionally, stable isotope analysis of specific amino acids can be more reliable in preserving the isotope values of the source [31].

### General inferences about feeding biology

Very little variability among tissue stable isotope values of individuals in a collection could suggest a feeding strategy in which either animals specialize on an isotopically consistent food source, or they consistently integrate an isotopically heterogeneous food source across their feeding range. Conversely, large variability among individuals in a collection could indicate that individuals specialize on different food sources or feed in isotopically distinct microhabitats in a heterogeneous “landscape” (for most species, the “landscape” would be a single vestimentiferan or mussel aggregation) [32,33]. Since we made many collections, we often had the advantage of observing whether a pattern or relationship in tissue stable isotope values is consistent in more than one collection.

**Figure 5:**
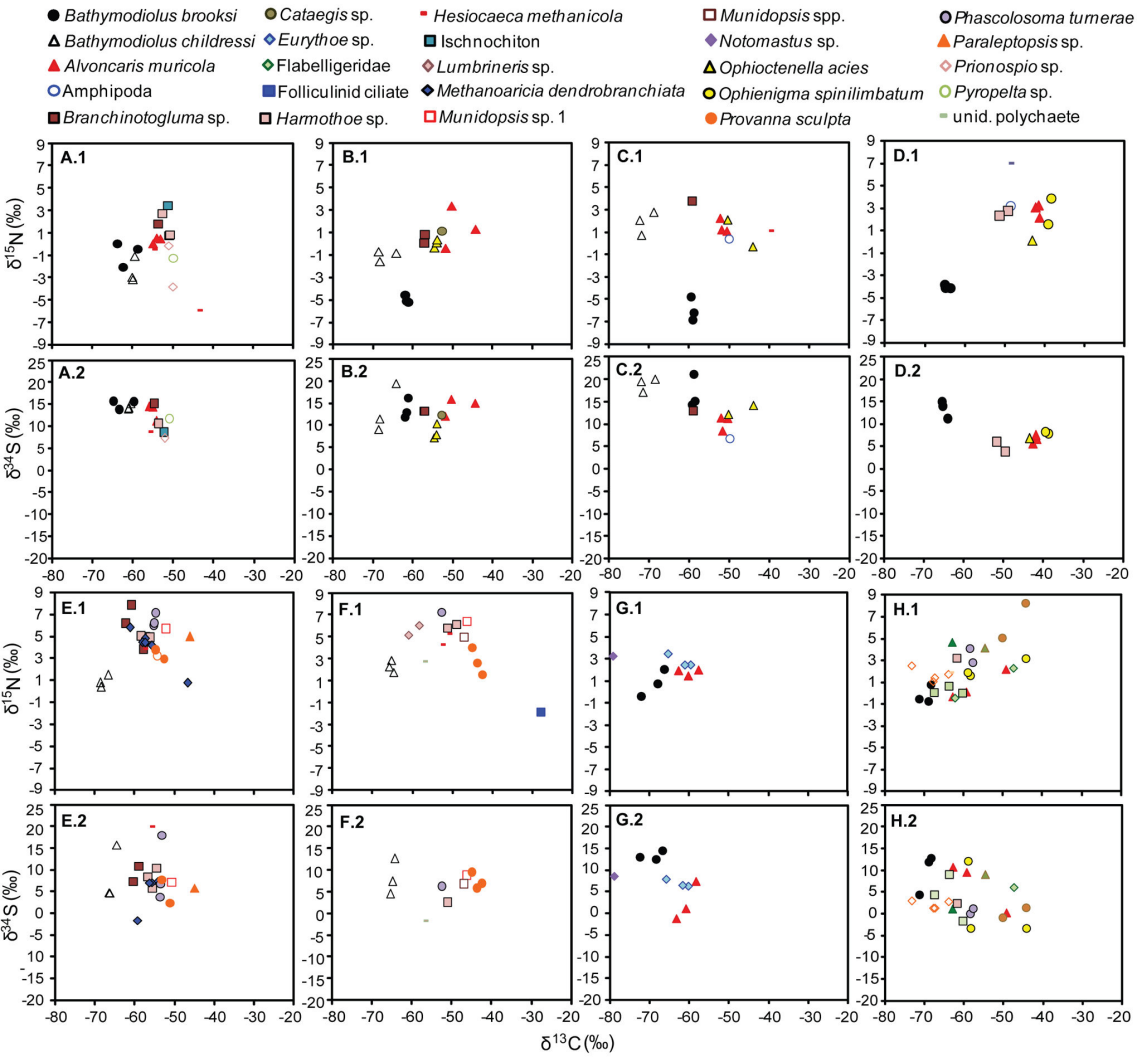
Stable isotope values for every individual sampled from the mussel collections from the Garden Banks, Green Canyon and Walker Ridge study sites (1200-1900m depth). (*X*.1) δ^15^N and (*X*.2) δ^34^S vs. δ^13^C, where *X* = a sequential letter representing a single collection. For example, A.1 and A.2 display the δ^15^N vs. δ^13^C and δ^34^S vs. δ^13^C, respectively, for one collection, B.1 and B.2, another, and so on. Different animal taxa are represented by different symbols. The site information, collection coordinates, and reference to the quantitative community study [2] are shown in Table **1**.

**Figure 6:**
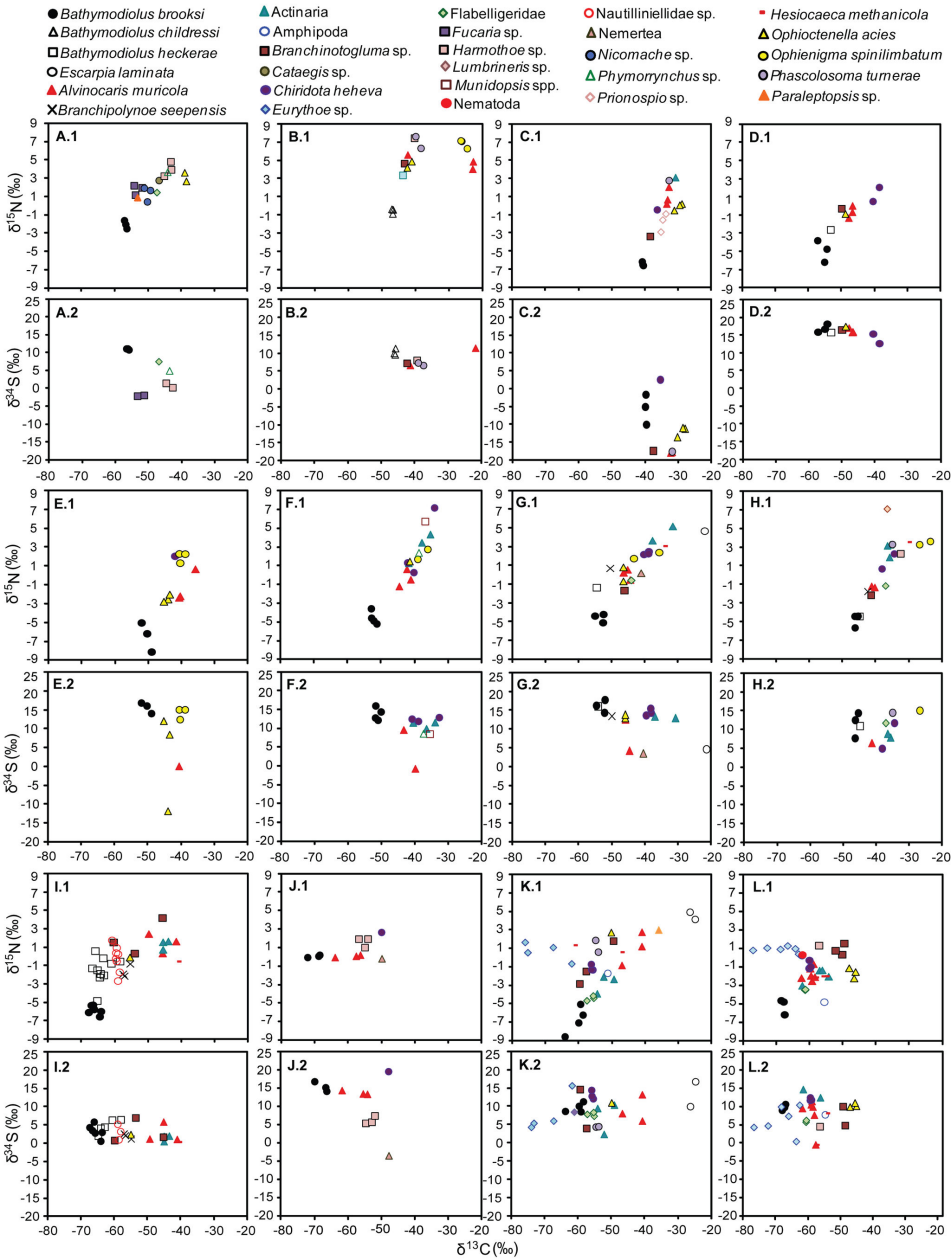
Stable isotope values for every individual sampled from mussel collections from the **Ala** minos Canyon and Atwater Valley study sites (2200-2800m depth). (*X*.1) δ^15^N and (*X*.2) δ^34^S vs. δ^13^C, where *X* = a sequential letter representing a single collection. For example, A.1 and A.2 display the δ^15^N vs. δ^13^C and δ^34^S vs. δ^13^C, respectively, for one collection, B.1 and B.2, another, and so on. Different animal taxa are represented by different symbols. The site information, collection coordinates, and reference to the quantitative community study [2] are shown in Table **1**.

**Figure 7:**
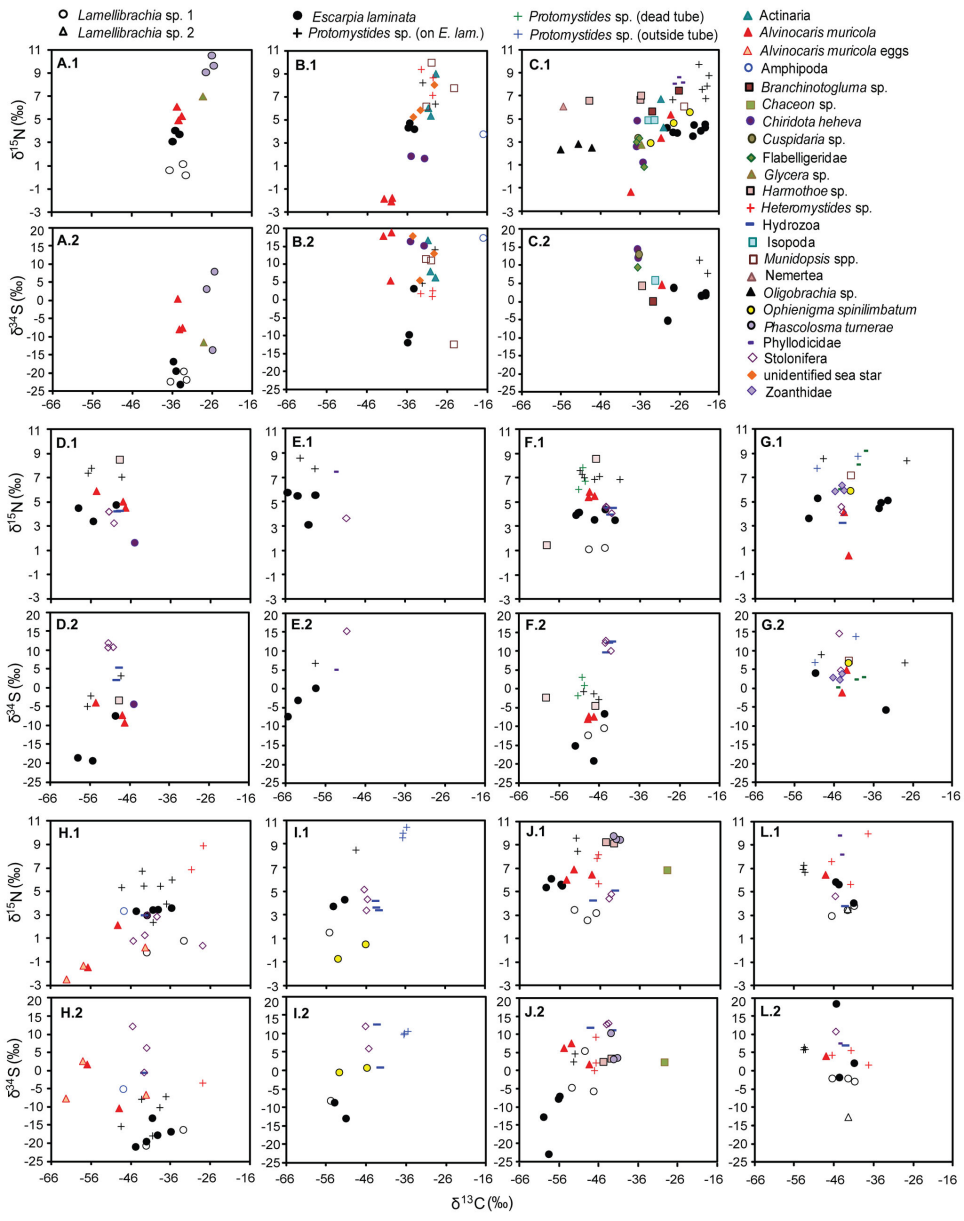
Stable isotope values for every individual sampled from vestimentiferan collections. (*X*.1) δ^15^N and (*X*.2) δ^34^S vs. δ^13^C, where *X* = a sequential letter representing a single collection. For example, A.1 and A.2 display the δ^15^N vs. δ^13^C and δ^34^S vs. δ^13^C, respectively, for one collection, B.1 and B.2, another, and so on. Different animal taxa are represented by different symbols. *Protomystides* sp. is split into three categories with different symbols representing the habitat the worms were found in. “*Protomystides* sp. (on *E. lam*)” represents individuals attached to the obturaculum of *Escarpia laminata*, “*Protomystides* sp. (dead tube)” refers to individuals collected inside empty vestimentiferan tubes, and “ *Protomystides* sp. (outside tube) refers to individuals attached to the outside of vestimentiferan tubes. In all cases, the worms had inhabited a thin flexible tube attached to a surface. The site information, collection coordinates, and reference to [2] quantitative community study are shown in Table **1**.

Taxa that have low variability in their stable isotope values within a collection are the brittle star *Ophioctenellaacies* (Figures **5B,C** and **6A,B,C,E,L**), the sipunculid *Phascolosoma* *turnerae* (Figure **5E,H**, Figure **6B,K** and Figure **7A,J**), the anemones (Actinaria; Figures **6F,G,H,I,K,L** and **7B,C**), and the polynoid polychaete *Harmothoe* sp. (Figure **5A,D,E,F**, Figure **6A,J** and Figure **7C,J**), although the two *Harmothoe* sp. individuals in one vestimentiferan collection had very different δ^13^C and δ^15^N values (Figures **7F**, **1**). There were many other examples like this for species only found in one collection. In fact, most species tended to group together within collections on plots of δ^15^N vs. δ^13^C and δ^34^S vs. δ^13^C (Figures **5**, **6**, and **7**).

### *Alvinocaris* *muricola*

The shrimp *Alvinocaris* *muricola* was one of the most common and numerically abundant animals in both mussel and vestimentiferan habitats [2]. *Alvinocaris* *muricola* ’s tissue stable isotope values were sometimes variable and sometimes similar to one another within collections (Figures **5**, **6**, and **7**). In vestimentiferan collections, *A* *. muricola* were often among the most depleted in δ^15^N relative to other animals in the same collections (Figure **7**), sometimes even more depleted than the vestimentiferans (Figure **7B,C,G**). In mussel collections, *A* *. muricola* were sometimes enriched and sometimes depleted in δ^15^N relative to other animals, but were always enriched relative to the mussels (Figures **5** and **6**). Some *A* *. muricola* individuals had δ^15^N compositions greater than 6‰, which is similar to the δ^15^N composition of surface-derived particulate organic matter (POM) [15], although these same individuals still had relatively depleted δ^13^C and δ^34^S compositions. The overall ranges for δ^13^C (-63.7 to -20.8‰) and δ^34^S (-18.5 to +19.1‰) in *A* *. muricola* could reflect a combination of seep- and surface-derived nutrition.

The variability in *A* *. muricola* ’s tissue stable isotope values suggests that individuals specialize on different food items or on the same food item in isotopically distinct microhabitats. The lowest δ^15^N values may reflect the shrimp grazing upon free-living bacteria that are fixing local inorganic nitrogen, and the enriched values may reflect some feeding upon animals at higher trophic levels, such as small predatory meiofauna, or consumption of surface-derived material. It is also plausible that individual shrimp specialize in isotopically distinct microhabitats as they are frequently observed near the tops of vestimentiferan tubes, but can also tolerate the chemical conditions near the sediment surface in vestimentiferan and mussel habitats.

### Hesiocaeca methanicola

**Figure 8:**
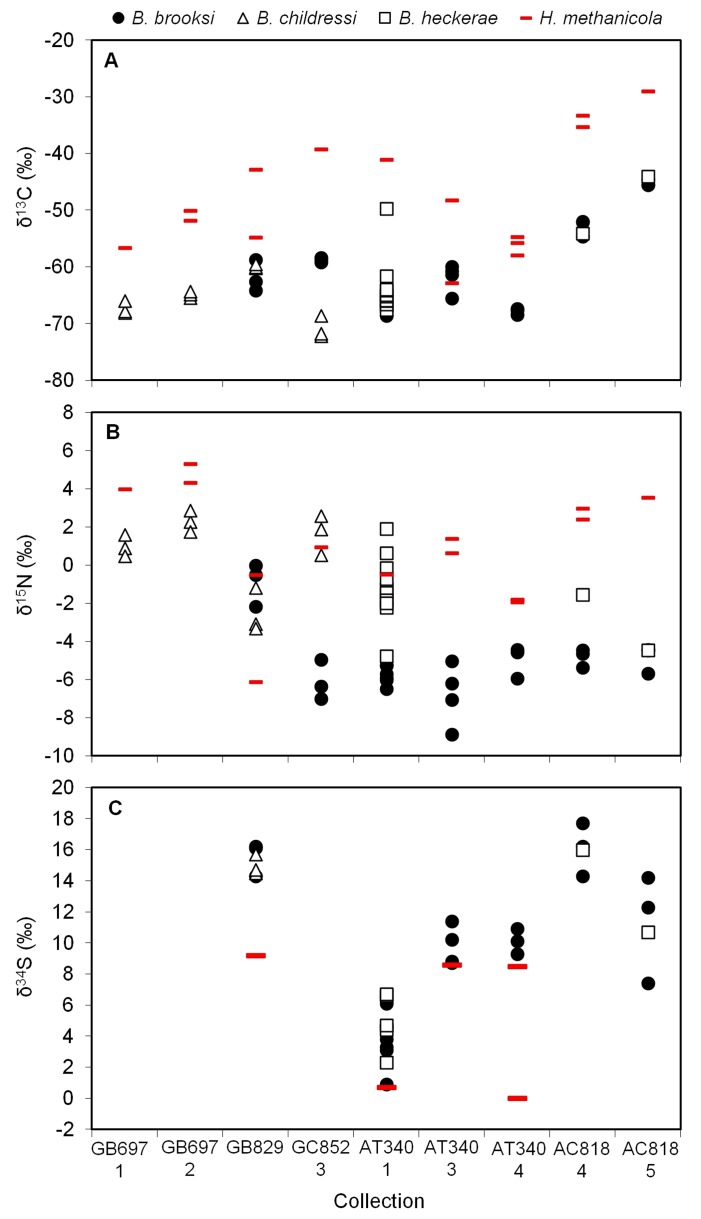
Tissue stable isotope values for the methane ice worm *Hesiocaecamenthanicola* and *Bathymodiolus* mussels separated by collection. (**A**) δ^13^C vs. collection, (**B**) δ^15^N vs. collection, and (**C**) δ^34^S vs. collection. Different symbols are used for different *Bathymodiolus* species.

Previous work has shown that the methane ice worm *Hesiocaecamethanicola* is a bacterivore, and therefore its tissue isotope values reflect the isotope compositions of the free-living microbial community [34]. In the previous study, the worms occupied depressions in a sulfide-containing methane hydrate and had tissue δ^13^C values of -24.7 to -23.8‰, δ^15^N of 5.3 to 6.3‰, and δ^34^S of 1.9 to 3.6‰. These values strongly indicated that the primary food source utilized by *H* *. methanicola* was not methanotrophic bacteria as originally hypothesized, but rather chemoautotrophic sulfur bacteria on the hydrates [34]. In mussel habitats in our study, *H* *. methanicola* δ^13^C values ranged from -62.9 to -29.0‰, δ^15^N ranged from -6.1 to +5.3‰, and δ^34^S from 0.7 to 20.8‰ (Figure **8**), which indicates large variability in the isotope compositions of the microbes on which they feed and suggests that they do not specialize on a single type of bacteria.

### Sediment-dwelling sipunculids and holothurians

The sediment-dwelling sipunculid *Phascolosoma* *turnerae* collected with vestimentiferans and mussels had enriched δ^15^N values relative to other animals in the same collections, but had δ^13^C and δ^34^S values that were quite depleted (δ^13^C = -58 to -30‰ and δ^34^S = -18.2 to +14.3‰) relative to surface POM (δ^13^C = -22 to -15‰ [11] and δ^34^S = 10 to 20‰ [5,35]) (Figures **5E,F,H**, **6B,C,H,K** and **7A,J**). In most of the collections, *P* *. turnerae* had δ^15^N values that were approximately 6-8‰ higher than the mussels or vestimentiferans with which they were collected. Previous work on the related species *Phascolosoma* *vulgare* showed that these sediment feeders demonstrate some specificity in the grain size of sediment they ingest and that fecal pellets account for 92% of this species’ caloric intake [36]. In a study of marine copepods, a major meiofaunal taxon in marine sediments, feces were enriched in δ^15^N by about 8‰ and δ^13^C by about 1‰ relative to their food source [37]. Thus, a diet primarily of fecal pellets is consistent with tissue stable isotope values that are enriched in δ^15^N while remaining relatively depleted in δ^13^C and δ^34^S. The sediment-feeding holothurian *Chiridotaheheva* was occasionally more enriched in δ^15^N than other animals in the same collections, including the predators, but not as consistently as *P* *. turnerae* (Figures **6C-H,J-L** and **7B,C,D**). *C* *. heheva* ’s diet may consist of a more variable mixture of fecal pellets, meiofauna, and sediment microbes than that of *P* *. turnerae* .

### Bristle worms

The most depleted δ^13^C value of all species in all collections was found in the bristle worms *Notomastus* sp. (-80.3‰) and *Eurythoe* sp. (several individuals ranged from -77.3 to -75.4‰ and max. -59.5‰) from mussel collections. These values were often more depleted than the δ^13^C values of the mussels with which they were collected (Figures **5G** and **6K,L**). *Notomastus* species in other ecosystems are sub-surface deposit feeders [38]. Since both methane and isotopically depleted DIC are more abundant below the sediment surface than above, the overall δ^13^C value of the food available to *Notomastus* sp. would be more depleted than the food available to surface-feeding animals. Other *Eurythoe* species are omnivorous and scavengers and can feed by extending their pharynx to feed under the sediment or to ingest larger food items such as dead animal tissue [39]. We cannot make a true inference about the food source of this *Eurythoe* species associated with bathymodiolin mussels, but its low δ^13^C values and high δ^15^N values in the two collections from AT340 stand apart from the almost linearly arranged δ^15^N vs. δ^13^C data points for the other fauna in these collections (Figure **6K,L**). This could be consistent with a scavenger or predatory lifestyle, possibly feeding on isotopically depleted organisms below the sediment surface.

### Suspension feeders

The suspension-feeding stoloniferans, hydroids, and zoanthids that colonize the tops of vestimentiferan tubes up to a meter above the sediment surface had δ^13^C values around -50‰, similar to most sediment-dwellers in the same collections (Figures **2** and **7**). Stoloniferans, hydroids, and zoanthids are all sessile cnidarians and feed on POM and small swimming animals that come in contact with their nematocysts. Because seep fluids escape from the sediment slowly and can be rapidly diluted by seawater, dissolved reduced chemicals from seep fluids are likely not present in sufficient concentrations at the plume level of vestimentiferan aggregations to fuel chemoautotrophy or influence the δ^13^C of DIC [21]. Therefore, it is more likely that organic material is produced via chemoautotrophy or methanotrophy in the sediment and subsequently transported to the tube tops. We did not sample meiofauna (animals between 63 µm and 1 mm), but they are a possible link between microbial primary production and heterotrophic macrofauna. Resuspension of isotopically depleted POM, including bacteria, from shallow sediments could also contribute to the depleted tissue isotope signal in the cnidarians.

### *Protomystides* “cap worms” on *Escarpia laminata* hosts

The phyllodocid polychaete *Protomystides* sp. was frequently found on top of the vestimentiferan *Escarpialaminata* (Figure **2**). The *Protomystides* sp. build a matrix of tubes, forming a casing (or “cap”) that affixes them to the surface of the obturaculum (the anterior-most part of the vestimentiferan). This casing can house more than 20 individual *Protomystides* sp. on a single *E* *. laminata* individual, many of which are tiny (personal observation). A blood-sucking (hematophagous) lifestyle was previously suggested for the related phyllodocid *Galyptomystides* *aristata* found in the Galapagos Rift and East Pacific Rise [40]. The guts of the *Protomystides* sp. in our collections contained a red substance, which, given the location of the polychaetes and the lifestyles of close relatives, was hypothesized to be vestimentiferan blood. If there is a parasitic relationship between *Protomystides* sp. and *E* *. laminata* , we would expect to see a correlation between the tissue stable isotope values of *Protomystides* sp. and the *E* *. laminata* individual upon which they were living.

**Figure 9:**
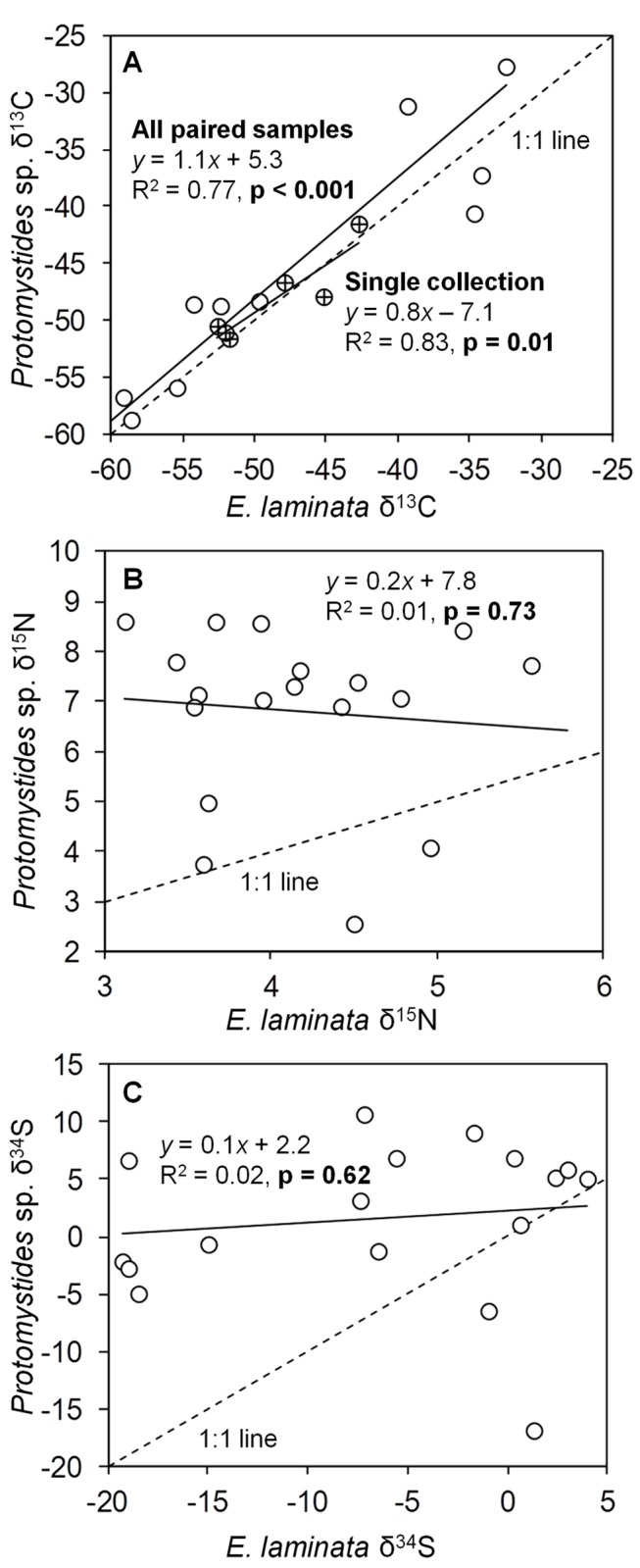
Tissue stable isotope values of paired *Protomystides* sp. and *E* *. laminata* . Tissue (**A**) δ^13^C, (**B**) δ^15^N, and (**C**) δ^34^S values in the small polychaete *Protomystides* sp. vs. the paired *Escarpialaminata* individual upon which the *Protomystides* sp. was found. The circles with crosses in panel (**A**) denote tissue δ^13^C values for paired samples of *Protomystides* sp. and *E* *. laminata* from a single collection from the AT340 study site. The solid lines denote best-fit regression lines; the top one is for all samples and the bottom line for the single collection.

There was a strong linear relationship between the tissue δ^13^C of *Protomystides* sp. and their paired *E* *. laminata* (p<0.001; R^2^=0.77; Figure **9A**). This relationship was not significant for tissue δ^15^N (p=0.73; R^2^=0.01; Figure **9B**) or δ^34^S (p=0.62; R^2^=0.02; Figure **9C**), but 14 out of 18 *Protomystides* sp. δ^15^N values were 2-5‰ higher than their paired *E* *. laminata* , consistent with trophic enrichment in tissue δ^15^N [29,41]. Since δ^13^C values tended to vary by collection, it was possible the apparent correlation simply reflected the use of the same carbon pool by both *Protomystides* sp. and *E* *. laminata* . To examine whether there was in fact an individual-level correlation, we examined a single collection that contained 6 paired *Protomystides* sp. and *E* *. laminata* samples. The total range in *E* *. laminata* δ^13^C values in this collection was -52.6 to -42.7‰, and the regression revealed a strong and significant correlation between paired individuals (R^2^=0.83, p=0.01; Figure **9A**). Whether or not *Protomystides* sp. feeds on vestimentiferan blood, these data suggest fidelity to the nutrition of a single *E* *. laminata* individual. Overall, the δ^13^C data suggest that *Protomystides* sp. obtains the bulk of its carbon requirements from *E* *. laminata* , but the δ^15^N and δ^34^S suggest that bulk nitrogen and sulfur demands may not be met by this nutritionally limited food source. It is also possible that the vestimentiferan soft tissue we sampled and the blood, which we did not sample, have similar δ^13^C but different δ^15^N and δ^34^S contents.

Some *Protomystides* sp. individuals also inhabited the outsides of vestimentiferan tubes or insides of empty tubes. The isotope compositions of these individuals were within the same range as those found on top of the live vestimentiferans (Figure **7F,G**), and therefore no apparent difference in isotopic niche from their counterparts atop *E* *. laminata* obturacula. The individuals found on the outsides of tubes were attached via the same casing we found on the tops of *E* *. laminata* obturacula, but these casings contained only 1-3 larger individuals and no tiny ones. The detailed morphology of the digestive system in the related vent species *G* *. aristata* showed the presence of a “septum” that could facilitate the long-term storage of a blood meal [40]. If these seep congeners share this adaptation, they may feed on vestimentiferan blood for the early stages of their life and then disperse to other locations at later stages, either feeding on stored blood or ingesting other materials. Another possibility is that blood is most important for females during reproduction and, like female mosquitoes, they need a continuous supply of blood before laying eggs. This could account for the large number of tiny worms present only in the obturacula casings.

#### Commensal polychaetes inside mussel and clam hosts

Another relationship that was of *a priori* interest was that of polychaetes living commensally within the gills of mussel and clam hosts. In a study of the commensal polynoid *Branchipolynoesymmytilida* living inside the body cavity of the hydrothermal vent mussel *Bathymodiolus* *thermophilus* , there was a strong correlation between the tissue isotope compositions of individual polynoids and their hosts for both δ^13^C and δ^15^N [16]. Furthermore, the average enrichment in *B* *. symmytilida* tissue δ^15^N relative to the host mussel tissue was 3.2‰, which is what is expected for single trophic level enrichment [29].

**Figure 10:**
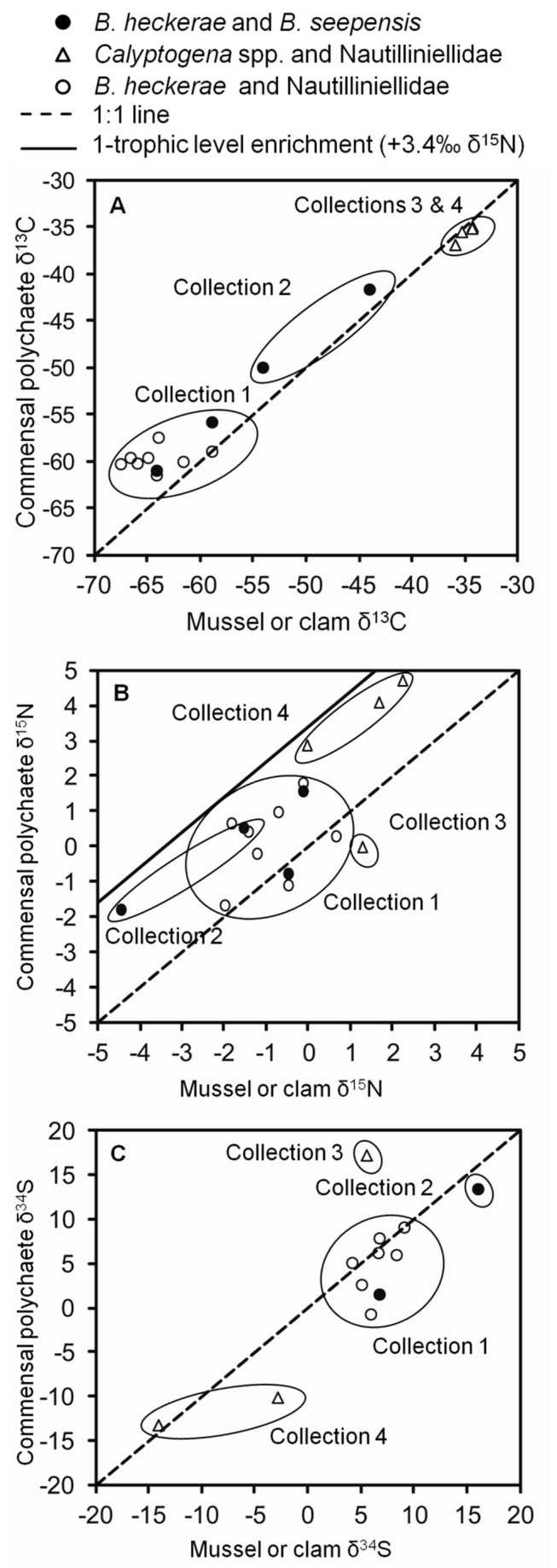
Paired commensal polychaetes and host symbiotic bivalves. Tissue (**A**) δ^13^C, (**B**) δ^15^N, and (**C**) δ^34^S values in the commensal polynoid *Branchipolynoeseepensis* or nautilliniellid polychaete vs. the paired *Bathymodiolusheckerae* *,* *B* *. childressi* , or vesicomyid clam ( *Calyptogena* *ponderosa* or an undescribed vesicomyid) individual within whose gills the polychaete was found. The ellipses shown in the figure encircle individuals collected in the same <1m^2^ area. There are fewer data for δ^34^S, because some samples did not have enough remaining material after δ^13^C and δ^15^N analysis.

In the present study, the polynoids *Branchipolynoeseepensis* were collected from inside the mantle cavity of *B* *. heckerae* and a species of nautilliniellid from inside the mantle cavity of *Bathymodiolusheckerae* , *Calyptogena* *ponderosa* and an undescribed vesicomyid. In general, δ^13^C and δ^34^S values were similar between the polychaetes and their bivalve hosts (Figure **10A,C**). However, the similarity in isotopes could simply be due to shared location as with other associated fauna. The linear relationship between paired *B* *. seepensis* and *B* *. heckerae* δ^13^C was the strongest evidence for a trophic relationship, because the 4 samples from 2 collections had enough variability to show a correlation (R^2^=0.99, p=0.003).

As is typical for seep animals, the overall variability in δ^15^N was much less than that of δ^13^C and δ^34^S, making correlations more difficult to discern. Overall, there seemed to be a positive relationship between the δ^15^N of the commensal polychaetes and their paired bivalve hosts, and differences between most of the polychaetes and hosts were between 3.4‰ and 0‰ (shown as most of the points lying between the 1:1 line and the line that signifies a 3.4‰ trophic enrichment; Figure **10B**). The δ^15^N values do not rule out a trophic relationship, but the small data set makes it difficult to discriminate trophic interaction from variation between local nitrogen pools. The nature of a trophic relationship between the polychaetes and bivalves could be partial predation of the bivalve tissue or consumption of a product such as mucus, gametes, or pseudofeces.

## Conclusion

The aim of this study was to elucidate trophic interactions in seep communities as part of a greater effort to understand how seeps function and how they tie into the functioning of the greater Gulf of Mexico ecosystem. Analysis of bulk tissue stable isotope content is a logical first step in this system given the efficiency and cost effectiveness of this method compared with others. This method alone, however, cannot reliably be used to infer trophic niches or food web structure until we attain a much greater understanding of the isotopic compositions of the inorganic sources and the abiotic and biotic processes that affect isotopic fractionation in seep environments. Even in more well-studied ecosystems, researchers have cautioned against applying data from different systems without experimental and environmental data from the system of interest [42], and some have shown using empirical data and modeling that isotopic and trophic niches do not always align (e.g. high isotopic variability among individuals does not necessarily imply individual specialization when dealing with an isotopically heterogeneous landscape [32,33]).

The data presented here and in related studies show very high spatial variability in tissue stable isotope contents between locations [8,17,20], and there is some indication from the current study that there is also micro-scale spatial heterogeneity in the isoscape of single aggregations, especially in vestimentiferan habitats, since their tall tubes create a structurally and chemically heterogeneous habitat.

In moving forward toward understanding stable isotopes and food webs in seep environments, a better understanding of the sources and processes affecting nitrogen isotopes at seeps is urgently needed, since spatial and taxonomic variability in tissue δ^15^N values preclude reliable assignment of trophic level based on bulk tissue δ^15^N content. Analyzing the nitrogen stable isotope content of specific amino acids could be very fruitful, since phenylalanine shows no change with trophic level, and therefore indicates the isotopic composition of nitrogen sources at the base of the food chain, while glutamic acid shows an increase of ~7‰ with each trophic level, allowing for definition of trophic level [31,43]. This method is considerably more expensive, but some researchers are already beginning to apply it to deep-sea food webs (http://www.jamstec.go.jp/biogeos/j/elhrp/isotope/index_e.html). Such an analysis could help us to better calibrate the bulk tissue isotope values presented in this study. Additionally, the missing links in the seep food web, namely bacteria and meiofauna, should be sampled from the local environments along with the macrofaunal community. Together with our data, these proposed analyses would help to build a more complete picture of hydrocarbon seep food webs in the Gulf of Mexico.

### Data availability

The original stable isotope data presented in this manuscript are available on the U.S. Geological Survey Ocean Biogeographic Information System found at http://www.usgs.gov/obis-usa/
